# Early Methylene Blue for Mortality Reduction in High-Dose VasoPREssor-Dependent Septic Shock (EMPRESS): protocol for a multicenter randomized controlled trial

**DOI:** 10.1186/s13049-026-01563-y

**Published:** 2026-01-24

**Authors:** Jing-chao Luo, Li Ma, Ming-hao Luo, Miguel Ibarra-Estrada, Pei-jing Yan, Zheng-ang Zhang, Mei Meng, Eduardo Kattan, Sen Lu, Chun Pan, Jean-Louis Teboul, Glenn Hernández, Xiao-bo Huang

**Affiliations:** 1 Department of Critical Care Medicine, Sichuan Academy of Medical Sciences and Sichuan Provincial People’s Hospital, University of Electronic Science and Technology of China, Chengdu, China; 2 Department of Critical Care Medicine, First People’s Hospital of Liangshan Yi Autonomous Prefecture, Xichang, China; 3 Medical Administration Department, Sichuan Academy of Medical Sciences and Sichuan Provincial People’s Hospital, University of Electronic Science and Technology of China, Chengdu, China; 4 Clinical Research Center, Sichuan Academy of Medical Sciences and Sichuan Provincial People’s Hospital, University of Electronic Science and Technology of China, Chengdu, China; 5 Department of Intensive Care Medicine, School of Medicine, Shanghai Ninth People’s Hospital, Shanghai Jiaotong University, Shanghai, China; 6 Nuffield Department of Population Health, University of Oxford, Oxford, UK; 7 Department of Anaesthetic and Critical Care, Harefield Hospital, Heart, Lung and Critical Care Group, Guy’s and St Thomas’ NHS Foundation Trust, London, UK; 8 Departamento de Medicina Intensiva, Facultad de Medicina, Pontificia Universidad Católica de Chile, Santiago, Chile; 9 The Latin American Intensive Care Network (LIVEN), Santiago, Chile; 10 Unidad de Terapia Intensiva, Hospital Civil Fray Antonio Alcalde, Universidad de Guadalajara, Guadalajara, Jalisco México; 11 Instituto Jalisciense de Cancerología. Guadalajara, Jalisco, Mexico; 12 Faculté de Médecine, Université Paris-Saclay, Le Kremlin-Bicêtre, France

**Keywords:** Septic shock, Methylene blue, Vasopressor, Mortality, Randomized controlled trial

## Abstract

**Background:**

Septic shock remains one of the most lethal emergency and critical care conditions, with underlying pathophysiology closely linked to uncontrolled vasodilation mediated by nitric oxide activation of the soluble guanylate cyclase pathway (NO–sGC–cGMP pathway). Methylene blue (MB), through its inhibition of this pathway, has demonstrated hemodynamic benefits and may serve as a targeted adjunctive therapy particularly in patients with septic shock requiring high-dose vasopressors, a severely vasoplegic subpopulation characterized by markedly elevated mortality. However, large-scale randomized controlled trials (RCTs) evaluating MB treatment for mortality benefit in high-dose vasopressor-dependent septic shock patients are currently lacking.

**Methods:**

This is an investigator-initiated, multicenter, open-label, blinded endpoint RCT that will recruit adult septic shock patients requiring norepinephrine equivalent doses > 0.3 μg/kg/min within 24 h of vasopressor initiation across multiple centers in China. A total of 566 patients will be randomized 1:1 to receive MB treatment (2 mg/kg loading dose followed by 0.25 mg/kg/h maintenance infusion for up to 48 h or 4 h after vasopressor discontinuation) or equal volume 5% dextrose control. Randomization will be stratified by baseline SOFA-1 score and study center. The primary endpoint is 28-day all-cause mortality. Secondary outcomes include ICU-free days, vasopressor-free days, ventilator-free days, in-hospital mortality, and SOFA score improvement.

**Discussion:**

This multicenter RCT will generate evidence on whether early MB administration reduces mortality in patients with high-dose vasopressor–dependent septic shock, potentially informing clinical practice regarding MB's role as an adjunctive therapy in severe septic shock management.

**Trial registration:**

ChiCTR2500112352.

## Introduction

Septic shock remains one of the most prevalent and lethal conditions in emergency departments and intensive care units (ICUs), with its underlying pathophysiology closely linked to nitric oxide (NO)-mediated uncontrolled vasodilation [1]. During septic shock, the systemic inflammatory response leads to endothelial dysfunction, where pro-inflammatory mediators activate nitric oxide synthases (eNOS and iNOS), resulting in excessive NO production. The excessive NO subsequently activates the soluble guanylate cyclase (sGC)-cyclic guanosine monophosphate (cGMP) pathway, causing vascular smooth muscle relaxation and reducing the vasoconstrictor response to both endogenous and exogenous catecholamines [1]. This severe vasodilation often necessitates high-dose vasopressor support, with increasing vasopressor requirements correlating with higher mortality risk [2]. While catecholamines, particularly norepinephrine, remain the first-line and mainstream vasopressors in septic shock [3], high-dose catecholamine administration not only exhibits diminishing efficacy but also leads to numerous adverse effects, including peripheral ischemia, arrhythmias, and increased myocardial oxygen consumption [4].

Methylene blue (MB), a sGC inhibitor, acts by binding to the heme–iron of sGC, thereby inhibiting the NO-sGC-cGMP pathway [5]. Since its first application in human septic shock in 1992 [6], multiple studies have demonstrated its hemodynamic benefits [7], with the first randomized controlled trial (RCT) by Kirov et al. in 2001 showing significant reduction in catecholamine requirements [8]. Pharmacokinetic studies indicate that MB distributes widely into body tissues, as evidenced by its large volume of distribution, and displays dose-dependent pharmacodynamic effects [9]. Retrospective data further suggest that a combined bolus-plus-maintenance regimen may be associated with improved survival compared with either strategy alone [10]. A recent single-center RCT also has highlighted the importance of early intervention, demonstrating improved outcomes including faster vasopressor weaning, reduced fluid balance, and shortened duration of mechanical ventilation and hospital stay, although the study was underpowered to detect mortality differences [11]. As a catecholamine-sparing agent, MB appears particularly advantageous in patients requiring high-dose vasopressors. This clinical population, primarily characterized by underlying vasoplegia, represents a more severe subpopulation with substantially higher mortality and has demonstrated greater responsiveness to MB in prior studies [10, 12]. However, large-scale RCTs evaluating early methylene blue intervention in refractory septic shock with high vasopressor requirements are still lacking.

## Methods/design

### Study objective

To evaluate whether early MB intervention significantly reduces 28-day mortality in septic shock patients requiring high-dose vasopressor support.

### Study design

**Fig. 1:**
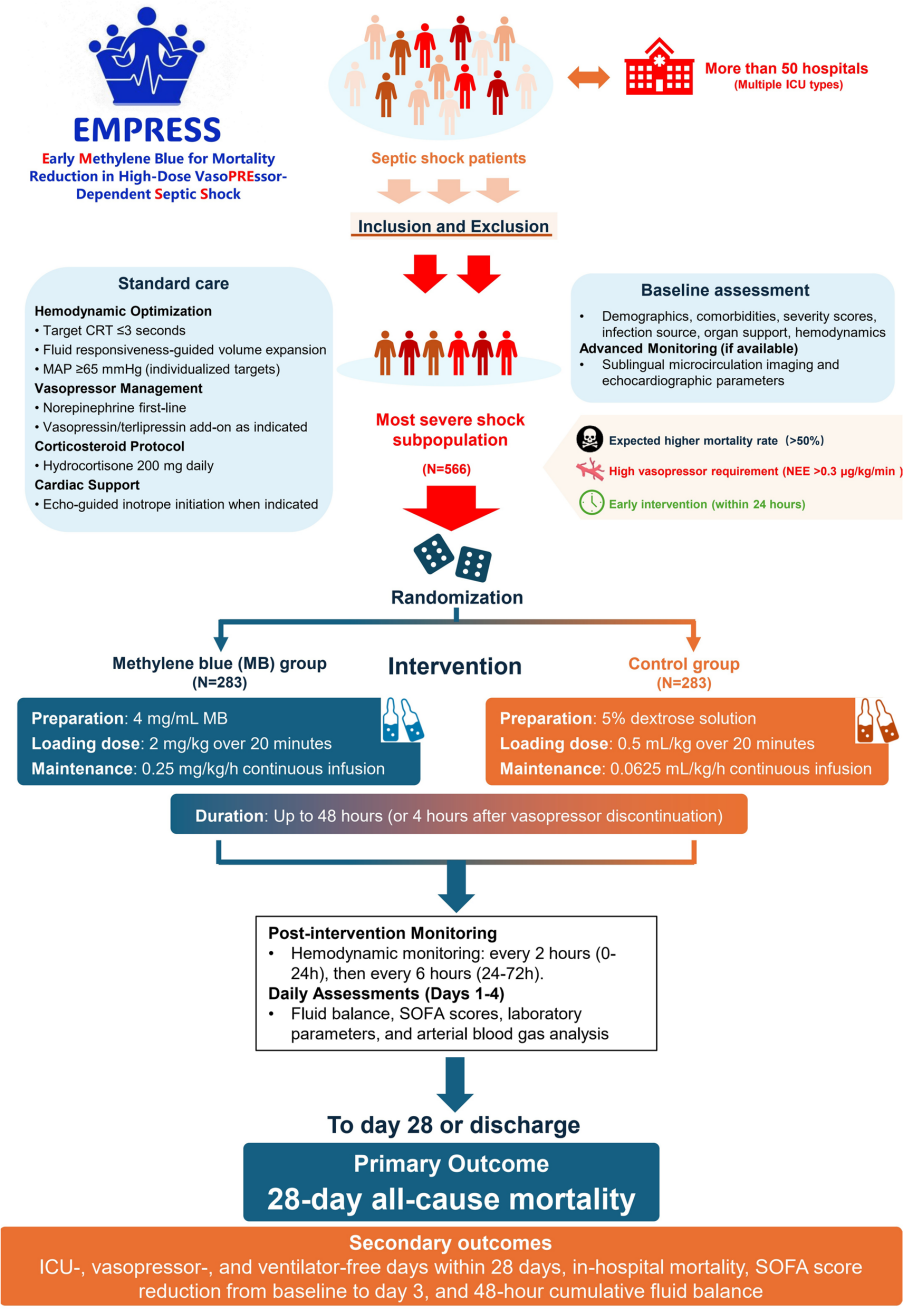
Study design and flow chart of the EMPRESS trial

This is an investigator-initiated, multicenter, prospective, parallel-group, open-label, blinded endpoint RCT (Fig. 1). This trial plans to recruit more than 50 centers (emergency, respiratory, medical, surgical, general, or other relevant ICUs) within mainland China, predominantly tertiary hospitals. The trial will be coordinated by Sichuan Academy of Medical Sciences and Sichuan Provincial People's Hospital, which act as the sponsors of the trial. The study received approval from the central ethics committee (approval number 2025–583), and the trial was registered in the Chinese Clinical Trial Registry (ChiCTR2500112352) on 12 November 2025.

### Study population

Patients with early septic shock requiring high-dose vasopressor support after initial resuscitation will be enrolled.

#### Inclusion criteria

1. Age ≥ 18 years
2. Septic shock as defined by Sepsis-3 criteria:
  - Suspected or documented infection
  - Requirement for norepinephrine to maintain mean arterial pressure (MAP) ≥ 65 mmHg
  - Blood lactate > 2 mmol/L despite adequate fluid resuscitation
3. Within 24 h of norepinephrine initiation
4. At enrollment screening, an equivalent norepinephrine dose exceeding 0.3 μg/kg/min sustained for at least 30 min.
  - The norepinephrine dose referenced here is calculated based on norepinephrine base, which corresponds to a norepinephrine bitartrate dose of 0.57 μg/kg/min as commonly used in clinical practice in China.
  - Norepinephrine base (μg/kg/min) = 0.53 × Norepinephrine bitartrate (μg/kg/min) + 1.0 × Epinephrine (μg/kg/min) + 0.01 × Dopamine (μg/kg/min) + 0.06 × Phenylephrine (μg/kg/min) + 2.5 × Vasopressin (U/min) + 0.0025 × Angiotensin II (ng/kg/min) + 10 × Terlipressin (μg/kg/min) + 0.125 × Metaraminol (μg/kg/min) [13–15]

#### Exclusion criteria

1. Pregnancy or lactation
2. Severe acute hypoxemic respiratory failure (PaO₂/FiO₂ < 100 mmHg)
3. Severe pulmonary arterial hypertension
4. Anticipated life expectancy < 48 h
5. Active non-distributive shock (hemorrhagic, obstructive, hypovolemic, or primary cardiogenic shock)
6. Pending damage control surgery
7. Extensive burns (> 20% total body surface area)
8. Glucose-6-phosphate dehydrogenase deficiency (self-reported or confirmed)
9. Known allergy to MB, phenothiazines, or food dyes
10. Recent use (within 4 weeks) of serotonergic agents (e.g., SSRIs, SNRIs, MAO inhibitors)
11. Declined consent by patient or legal surrogate
12. Prior administration of MB during the current disease episode

#### Withdrawal criteria

1. ​Occurrence of serious adverse events (SAEs) potentially related to study intervention
2. Withdrawal of consent by patient or legal representative
3. Patients mistakenly included due to human error who did not meet the inclusion criteria
4. Investigator determination that continued participation may compromise patient safety
5. Initiation of mechanical circulatory support for objective clinical reasons

#### Serious adverse event management procedures

1. SAEs must be reported to the Data Safety Monitoring Board (DSMB) and sponsor within 24 h
2. All SAEs will be managed according to the predetermined safety management protocol specified in the ethics committee application
3. Documentation includes causality assessment and follow-up until resolution

### Randomization

Randomization will be conducted via a centralized, web-based system with 1:1 allocation. Stratification factors include baseline Sequential Organ Failure Assessment score (based on SOFA-1, with subsequent assessments including both SOFA-1 and SOFA-2) [16, 17] (> 10 vs. ≤ 10) and study center. A permuted block randomization scheme (blocks of sizes 2, 4, or 6) will be used to ensure balanced group allocation and minimize prediction of assignment.

### Blinding

Given that MB causes visible blue discoloration of mucous membranes and urine, complete blinding of healthcare providers and patients is not feasible. Nevertheless, outcome assessors will be blinded to treatment allocation and will not participate in clinical care.

### Intervention protocol

#### MB group

- Drug preparation: 10 ampoules of MB injection (2 mL, 20 mg each) diluted with 5% dextrose solution [11] to achieve 4 mg/mL concentration (50 mL syringe)
- Loading dose: 2 mg/kg administered via infusion pump over 20 min (i.e., weight ÷ 2 mL)
- Maintenance dose: 0.25 mg/kg/h continuous infusion (i.e., weight ÷ 16 mL/h)
- Duration: Up to 48 h, or until 4 h after discontinuation of all vasopressors, whichever occurs first
- Treatment may be terminated if severe adverse reactions occur

#### Control group

- Equal volume of 5% dextrose solution
- Infusion protocol (loading and maintenance) identical to intervention group in terms of rate and duration

### Recommendation for concurrent treatment

Considering that patients in this study are in the early optimization phase following initial resuscitation, we provide the following recommendations for background care based on Surviving Sepsis Campaign 2021 [18], Japanese 2024 [19], and European Society of Intensive Care Medicine 2025 [20] guidelines. Patients are recommended to target tissue perfusion normalization (i.e., capillary refill time [CRT] ≤ 3 s) with continuous monitoring to guide circulatory optimization. Volume expansion is recommended to be guided by fluid responsiveness assessment through dynamic tests (passive leg raising test, end-expiratory occlusion test, or Trendelenburg position) or hemodynamic parameters (stroke volume variation, pulse pressure variation), with balanced crystalloids as the preferred choice. MAP is recommended to be maintained ≥ 65 mmHg, though higher MAP targets may be considered in only patients with chronic hypertension who demonstrate clinical improvement at elevated blood pressure levels. Regarding vasopressor management, norepinephrine remains the first-line agent, with catecholamine-sparing drugs (including vasopressin, terlipressin [avoiding high doses to reduce digital ischemia risk] [21], or angiotensin) added based on clinical indications. Hydrocortisone 200 mg/day is recommended for all patients and should be discontinued within 6 h of vasopressor cessation. Initiation of inotropic support is recommended based on objective evidence from echocardiographic assessment (emphasizing velocity–time integral [VTI] and left ventricular ejection fraction [LVEF]) or hemodynamic monitoring. Other treatment recommendations follow the latest guideline recommendations with unified training provided in a timely manner.

### Data collection

Patients will be followed for 28 days or until death, whichever occurs first. Table 1 shows details of the timeline of major parameters assessment. At baseline, demographic characteristics (age, sex, weight), comorbidities (Charlson Comorbidity Index), patient types (medical, surgical, and whether patients have undergone source control surgery), disease severity scores [Acute Physiology and Chronic Health Evaluation II (APACHE II), SOFA score [17]], infection source, respiratory and renal support modalities (mechanical ventilation and renal replacement therapy [RRT]), and hemodynamic parameters (systolic blood pressure [SBP], ​diastolic blood pressure [DBP], ​MAP, ​heart rate [HR], ​central venous pressure [CVP], lactate, CRT), will be recorded. If sublingual microcirculation monitoring equipment and qualified bedside ultrasound physicians are available at the center, sublingual microcirculation images will be captured and relevant echocardiographic parameters (including VTI and LVEF) will be calculated before medication administration. Vasopressor doses will be standardized as norepinephrine equivalents (μg/kg/min). Post-intervention, hemodynamic variables (SBP, DBP, MAP, HR, CVP) and vasopressor requirements will be monitored at 2-h intervals for the first 24 h, then at 6-h intervals from 24 to 72 h. Daily assessments during the initial 4 days will include fluid balance (including intake and output), SOFA score, and laboratory parameters: complete blood count (hemoglobin, white blood cells, platelets], coagulation profile (prothrombin time [PT], ​activated partial thromboplastin time [APTT], ​international normalized ratio [INR]), renal and hepatic function (creatinine, blood urea nitrogen [BUN, also converted to urea], total bilirubin [TB]), and ​arterial blood gas (ABG) analysis (lactate, ​PaO₂/FiO₂ ratio, and methemoglobin saturation [MetHb] if available). Additionally, we will also record the specific time points of vasopressor initiation, randomization, and intervention implementation, to determine the critical time intervals. Outcome measures include 28-day and hospital mortalities, ICU and hospital length of stay, duration of mechanical ventilation, and RRT utilization rate and duration. For selected centers, we will also include electrical impedance tomography as optional parameters before and after intervention. Trained research personnel will document all data using standardized electronic case report forms (eCRFs).

**Table 1:** Timeline of major parameters assessment

Category	Parameters	Baseline	Post-Intervention		
			First 72 h or 4 Days		Discharge/Death
Demographics	Age, Sex, Weight	√	×		×
Comorbidities	Pre-existing conditions (e.g., HTN, DM)	√	×		×
Infection Source	e.g., pulmonary, abdominal	√	×		×
Disease Severity	APACHE II Score	√	×		×
	SOFA Score (SOFA-1 & SOFA-2)	√	Daily, Day 1–4		×
Support Modalities	Respiratory modality, RRT	√	Daily, Day 1–4		√
Hemodynamics	SBP, DBP, MAP, HR, CVP	√	2 h (0–24 h), 6 h (24–72 h)		×
	CRT	√	×		×
Vasopressor Dose	Norepinephrine base equivalents (μg/kg/min)	√	2 h (0–24 h), 6 h (24–72 h)		×
Fluid Balance	Daily cumulative fluid intake/output	×	Daily, Day 1–4		×
Laboratory	Hemoglobin, WBC, Platelets	√	Daily, Day 1–4		×
	Creatinine, BUN, TB	√	Daily, Day 1–4		×
	Coagulation profile (PT, APTT, INR)	√	Daily, Day 1–4		×
ABG Analysis	Lactate, PaO₂/FiO₂ ratio, MetHb	√	Daily, Day 1–4		×
Outcomes	28-day and Hospital Mortality, LOICU, LOHS, MV Duration	×	×		√

*HTN* Hypertension, *DM* Diabetes mellitus, *APACHE* Acute Physiology and Chronic Health Evaluation, *SOFA* Sequential Organ Failure Assessment, *RRT* Renal replacement therapy, *SBP* Systolic blood pressure, *DBP* Diastolic blood pressure, *MAP* Mean arterial pressure, *HR* Heart rate, *CVP* Central venous pressure, *CRT* Capillary refill time, *WBC* White blood cell, *BUN* Blood urea nitrogen, *TB* Total bilirubin, *ABG* Arterial blood gas, *PaO₂* Partial pressure of arterial oxygen, *FiO₂* Fraction of inspired oxygen, *MetHb* Methemoglobin, *LOICU* Length of ICU stay, *LOHS* Length of hospital stay, *MV* Mechanical ventilation

### Clinical outcome assessment

#### Primary outcome

- 28-day all-cause mortality

#### Secondary outcomes

- ICU-free days within 28 days
- Vasopressor-free days within 28 days
- Ventilator-free days within 28 days
- In-hospital mortality
- Absolute reduction in SOFA scores from baseline to day 3 post-intervention
- 48-h cumulative fluid balance

(All support-free days are calculated within a 28-day observation period, defined as 28 minus the number of days requiring the respective support for survivors, and assigned a value of 0 for patients who die within 28 days).

#### Safety endpoints

- Methemoglobinemia: Defined as peak daily MetHb ≥ 5% on ABG analysis.
- Hemolysis is suspected when soy sauce-colored urine is observed along with a clinically significant decrease in hemoglobin and red blood cell count, accompanied by elevated lactate dehydrogenase and bilirubin levels. The diagnosis is confirmed through examination of red cell fragments and measurement of free plasma hemoglobin) levels exceeding 200 mg/L.
- Absolute difference in PaO₂/FiO₂ ratio between baseline and 48 h post-intervention.

### Sample size calculation

A meta-analysis integrating data from multiple Chinese studies on septic shock reported a 28 to 30-day mortality rate of 35.9% [22]. Previous studies have demonstrated that patients requiring high-dose norepinephrine base (> 0.3 μg/kg/min) experience approximately 1.5-fold higher mortality compared to overall patients [23, 24]. Therefore, we estimated the baseline 28-day mortality rate in our target population at 50% (calculated as 35.9% × 1.5, conservatively rounded). A single-center randomized controlled trial by Ibarra-Estrada et al. demonstrated that MB reduced 28-day mortality in septic shock patients from 46 to 33% (relative reduction of 28%) [11]; considering inter-study heterogeneity, we conservatively anticipated a 25% relative risk reduction in our study population (although patients requiring higher vasopressor doses demonstrate better response to MB [12]). Regarding stratification factors, we projected a 6:4 ratio between patients with SOFA-1 scores > 10 versus ≤ 10 based on the Ibarra-Estrada study data in high-dose norepinephrine patients (median SOFA-1 score 11 [IQR: 9–12]), while the ANDROMEDA-shock study [25] demonstrated that in the high-dose norepinephrine subgroup, those with SOFA-1 scores > 10 also exhibited a 1.5-fold higher 28-day mortality rate. Based on the overall mortality rate of 50% and these proportional relationships, we estimated 28-day mortality rates of 58% and 39% for patients with SOFA-1 scores > 10 and ≤ 10, respectively. Using a two-sided significance level (α) of 0.05 and power (1-β) of 0.8, the calculated sample size required 254 subjects per group. Accounting for an anticipated 10% dropout rate, the final total sample size was determined to be 566 patients (283 per group).

### Data quality monitoring and missing data management

The data quality monitoring mechanism operates under a comprehensive framework with clearly defined responsibilities across multiple domains. The Data Monitoring Committee, consisting of statistical experts and clinical investigators, oversees overall data quality through three major components. First, real-time logical validation in eCRF implements automated alerts for physiologically implausible ranges through extreme value detection, mandatory completion of critical variables (such as SOFA-1 subscores and vasopressor equivalents) with system-level lockout for missing fields, and dual independent entry with cross-verification protocols specifically for hemodynamic parameters and outcome assessments. Second, central statistical monitoring conducts regular systematic reviews of data patterns and potential anomalies, with predefined triggers (including significant protocol violations, data inconsistencies, or safety signals) for additional investigation or on-site visits. Third, to ensure on-ground quality control, we have established regional oversight through our long-term collaborative partners across different regions of China to conduct on-site audits and quality assessments. Beyond monitoring, regarding missing data management, distinct strategies will be employed based on data types and missingness mechanisms. Primary and secondary outcome measures require complete data collection from all participating centers with no imputation performed, thereby ensuring endpoint integrity. However, for baseline covariates and general parameters, missing data patterns will be assessed, with data missing at random (MAR) handled using multiple imputation by chained equations (MICE) with 50 iterations, while data missing completely at random (MCAR) may utilize complete case analysis as a sensitivity approach. Finally, comprehensive sensitivity analyses will be conducted under different missingness assumptions to evaluate result robustness across varying scenarios.

### Interim analysis

Two formal interim analysis meetings will be conducted by the DSMB via teleconference (or face-to-face, if possible) to review data relating to treatment efficacy, patient safety, and quality of trial conduct. The analyses will encompass the evaluation of conditional power for the primary endpoint, safety outcomes, and protocol adherence metrics. A recommendation to discontinue prematurely will be based upon there being clear evidence that MB provides protection or causes harm for an important clinical outcome. The DSMB will work on the principle that a difference of at least 3 standard errors in an interim analysis of a major outcome event (e.g., 28-day mortality) between the MB and control groups will justify halting, or modifying the study, before the planned completion of recruitment. Given the minimal impact of this approach on the type-I error rate, no adjustment is made to the final significance level.

### Statistical analysis

#### Analysis populations and data presentation

The primary analysis will be conducted on the modified intention-to-treat (mITT) population, which includes all randomized patients who received at least one dose of study intervention and had at least one post-baseline assessment. The mITT approach preserves randomization benefits while excluding patients who never received treatment, thereby reducing dilution effects. Complete ITT and per-protocol populations will serve as sensitivity analyses to ensure result robustness across multiple analytical populations. Continuous variables will be presented as mean ± SD or median (IQR) based on normality tests; categorical variables as frequencies (percentages).

#### Primary outcome analysis

The primary endpoint (28-day mortality) will be analyzed using absolute risk difference as the primary effect measure, which corresponds directly with sample size calculations (50% vs 37.5%) and provides strong clinical interpretability. Analysis includes: (1) Unadjusted absolute risk differences with 95% CI using chi-square or Fisher's exact test; (2) Adjusted odds ratios from logistic regression controlling for baseline APACHE II score, SOFA-1 score, age, baseline vasopressor dose, and other relevant covariates to effectively control for potential confounding from baseline imbalances. Both unadjusted absolute differences and adjusted OR will be reported, with absolute difference serving as the primary inferential measure given that critically ill populations have high risk of baseline differences requiring robust adjustment strategies.

#### Secondary outcomes and subgroup analyses

Secondary outcomes will be evaluated using appropriate parametric or non-parametric tests with similar adjustment strategies for time-related outcomes. Exploratory pre-specified subgroup analyses will examine treatment effects across demographic characteristics (age, gender), disease severity (SOFA-1 > 10 vs ≤ 10, APACHE II > 25 vs ≤ 25), treatment-related factors (baseline vasopressor dose, timing), and infection characteristics to identify patient populations most likely to benefit from intervention. Interaction tests will assess treatment effect heterogeneity across these subgroups, with the understanding that the absence of statistically significant interactions does not exclude the possibility of clinically relevant effect modification. Survival curve visualization will employ Kaplan–Meier methods with log-rank tests to provide intuitive survival trend visualization and evaluate treatment effect consistency over time.

#### Statistical methodology and sensitivity analyses

All tests will be two-sided with α = 0.05, following standard clinical trial methodology. Sample size calculations based on mortality differences (50% vs 37.5% mortality) provide 80% power, maintained in adjusted analyses given that variance inflation from covariate adjustment typically remains < 2.0, not significantly affecting statistical power. Multiple sensitivity analyses will ensure result robustness, including complete ITT analysis, per-protocol analysis, multiple imputation for missing data, multilevel modeling for center effects, and extreme case scenarios for lost follow-up. Interim analyses will use pre-specified alpha-spending functions to control type I error across multiple looks. All analyses will be performed using R and SAS software.

## Discussion

This protocol describes a multicenter, open-label RCT evaluating early MB intervention in septic shock patients requiring high-dose vasopressor support. With a planned enrollment of 566 patients, this represents the first and largest RCT of MB in this specific patient population.

To date, evidence supporting mortality benefit of MB comes from several meta-analyses [26–28], with inconsistent results. Some have shown that improvement in mortality while others lack statistical significance in patients with septic or distributive shock. Moreover, previous meta-analyses have largely relied on small, single-center RCTs or observational studies with substantial heterogeneity in patient characteristics, shock severity, timing of intervention, and dosing strategies. Such variability raises methodological concerns, as combining heterogeneous or lower-quality data could lead to misleading pooled estimates. Therefore, rigorously designed multicenter prospective RCTs remain essential to establish whether MB provides a definitive mortality benefit in septic shock.

This study specifically enrolls patients receiving norepinephrine for less than 24 h at a dose equivalent to more than 0.3 µg/kg/min for at least 30 min. This differs from previous studies [8, 11, 29], which allowed longer time windows and did not require a defined vasopressor threshold. The rationale for this criterion is twofold. First, while the optimal therapeutic window for MB in humans remains to be clearly established, preclinical data suggest early intervention may be more effective (with only one study indicating an 8-h window in murine models [29], Additionally, the 2023 RCT demonstrated that MB administration within 24 h of vasopressor initiation improved hemodynamics [11]. Therefore, administering MB early within this timeframe may target the critical period when therapeutic benefits are most likely to be observed. Second, patients requiring higher vasopressor doses and demonstrating worse hemodynamic profiles have been shown to exhibit greater activation of the NO–sGC–cGMP pathway and are therefore more likely to benefit from MB [12, 30]. Excluding patients on only minimal norepinephrine support also reinforces the role of MB as an adjunctive therapy, minimizing the dilution of treatment effect. In contrast, another ongoing Chinese trial (NCT06481410) uses a lower threshold of 0.1 μg/kg/min, which may include patients with mild circulatory dysfunction who could stabilize with conventional resuscitation alone, potentially requiring over 1,000 patients to detect mortality differences based on existing data [11].

The study adopts a loading-plus-maintenance regimen, in contrast to prior studies such as Ibarra-Estrada et al. [11], which administered MB as repeated daily infusions without a bolus. The rationale is threefold: (a) a loading dose provides more rapid inhibition of the NO–sGC–cGMP pathway, offering earlier hemodynamic rescue [31]; (b) small pilot studies [32, 33] have demonstrated that hemodynamic improvements, such as increased systemic vascular resistance index (SVRI), occur within one hour of a bolus; and (c) retrospective analyses have suggested that bolus plus continuous infusion is independently associated with improved 28-day survival [10].

The primary outcome is 28-day all-cause mortality, an objective and clinically meaningful endpoint that has not been adequately assessed in previous MB trials. Earlier studies [8, 11] primarily evaluated intermediate outcomes such as hemodynamic improvement or vasopressor weaning and were typically underpowered to detect mortality effects. These outcomes, especially weaning from vasopressors could be clinician-dependent. Because MB changes urine color, full double-blinding is challenging; however, the use of mortality as an objective endpoint reduces the risk of bias. A moderate effect of 25% relative risk reduction was estimated when calculating sample sizes based on previous studies.

Finally, known adverse effects of MB include impaired oxygenation, dose-related toxicity, and methemoglobinemia. For safety considerations, these risks have been incorporated into the trial design. All patients will undergo routine ABG analysis to monitor oxygenation and methemoglobin levels. Previous reports have suggested that MB infusion may be associated with oxygenation deterioration [9, 34]. Patients with baseline PaO₂/FiO₂ ratios below 100 mmHg may have limited tolerance for this potential risk and may also progress to venovenous extracorporeal membrane oxygenation support, prone positioning, or inhaled NO therapy, all of which could introduce confounding hemodynamic effects that we aim to avoid. Although a loading dose of 2 mg/kg is employed, adverse effects are generally rare below this threshold. All adverse events will be systematically recorded, and an independent data monitoring committee will review safety data at regular intervals.

Several limitations must be acknowledged. First, the open-label design may introduce bias, as treating clinicians will be aware of allocation; however, this will be mitigated by selecting objective primary outcomes and blinding outcome assessors and statisticians. Second, despite the aim of initiating treatment within 24 h of vasopressor use, delays in resuscitation prior to ICU transfer may occur, particularly as many participating centers are tertiary referral hospitals. Third, the trial does not mandate advanced hemodynamic monitoring (e.g., echocardiography or invasive devices) to minimize workload at recruiting centers and reduce missing data. Finally, biological samples for biomarker studies will not be collected due to logistical constraints.

## Conclusion

This multicenter RCT will provide novel evidence to determine whether early MB intervention provides mortality or other clinical benefits to septic shock patients requiring high-dose vasopressor support. If successfully completed, this study will inform evidence-based therapeutic decisions for this critically ill population with limited therapeutic options.

## Data Availability

Reasonable requests should be directed to the corresponding author for approval.

## Abbreviations

ABG: Arterial blood gas
APACHE II: Acute Physiology and Chronic Health Evaluation II
APTT: Activated partial thromboplastin time
BUN: Blood urea nitrogen
CBC: Complete blood count
cGMP: Cyclic guanosine monophosphate
CRT: Capillary refill time
CVP: Central venous pressure
DBP: Diastolic blood pressure
DSMB: Data Safety Monitoring Board
eCRF: Electronic case report form
eNOS: Endothelial nitric oxide synthase
fHb: Free plasma hemoglobin
HR: Heart rate
ICU: Intensive care unit
iNOS: Inducible nitric oxide synthase
INR: International normalized ratio
IQR: Interquartile range
ITT: Intention-to-treat
LDH: Lactate dehydrogenase
LVEF: Left ventricular ejection fraction
MAP: Mean arterial pressure
MAO: Monoamine oxidase
MB: Methylene blue
MetHb: Methemoglobin saturation
MICE: Multiple imputation by chained equations
mITT: Modified intention-to-treat
NO: Nitric oxide
PaO₂: Partial pressure of oxygen in arterial blood
PT: Prothrombin time
RCT: Randomized controlled trial
RRT: Renal replacement therapy
SAE: Serious adverse event
SBP: Systolic blood pressure
SD: Standard deviation
sGC: Soluble guanylate cyclase
SNRIs: Serotonin-norepinephrine reuptake inhibitors
SOFA: Sequential Organ Failure Assessment
SSC: Surviving Sepsis Campaign
SSRIs: Selective serotonin reuptake inhibitors
SVRI: Systemic vascular resistance index
TB: Total bilirubin
VTI: Velocity-time integral
WBC: White blood cells
